# Expanding the genetic landscape of *SLC4A1*-linked hereditary spherocytosis: discovery of a novel TM9 variant using high-resolution genomic profiling analysis

**DOI:** 10.3389/fgene.2026.1831354

**Published:** 2026-08-27

**Authors:** Tejashree Anil More, Prabhakar S. Kedar

**Affiliations:** Department of Haematogenetics, ICMR-National Institute of Immunohaematology, Parel, Mumbai, India

**Keywords:** band 3, distal renal tubular acidosis, genotype–phenotype correlation, hereditary spherocytosis, next-generation sequencing, red cell membrane disorders, SLC4A1

## Abstract

**Introduction:**

Hereditary spherocytosis (HS) is the most common inherited red cell membranopathy caused by defects in erythrocyte membrane and cytoskeletal proteins, including ankyrin, spectrin, band 3, and protein 4.2. Among these, mutations in SLC4A1, which encodes the erythrocyte anion exchanger band 3 (AE1), account for approximately 20–30% of HS cases and it is associated with distal renal tubular acidosis (dRTA), reflecting phenotypic and functional heterogeneity.

**Methods:**

In this study, seven unrelated Indian patients with clinically suspected HS were investigated using detailed hematological, biochemical, and clinical evaluations along with eosin-5′-maleimide (EMA) binding assays. Molecular analysis was performed using targeted next-generation sequencing (t-NGS) covering 81 genes associated with red cell disorders, and the identified SLC4A1 variants were validated by Sanger sequencing. The structural and functional consequences of the variants were assessed through in silico tools including DynaMut, PolyPhen-2, and SIFT.

**Results:**

Seven SLC4A1 variants were identified, including six previously reported variants and one novel variant, p.Phe702Ser, detected in heterozygous or compound heterozygous states. These variants were distributed across both cytoplasmic and transmembrane domains of the band 3 protein. Most patients presented with mild to moderate HS characterized by anemia, jaundice, splenomegaly, reticulocytosis, and reduced EMA fluorescence. One patient harboring compound heterozygous variants (p.Arg490His and p.Ala858Asp) exhibited HS associated with dRTA, highlighting the functional diversity of SLC4A1 mutations. The novel p.Phe702Ser variant, located in the transmembrane domain TM9, was predicted to destabilize AE1 structure and potentially impair anion transport.

**Discussion:**

Marked intrafamilial phenotypic variability was observed despite identical genotypes. These findings expand the mutational spectrum of SLC4A1-related HS in Indian patients.

## Introduction

The red cell membrane consists of a lipid bilayer anchored to a cytoskeleton through protein complexes, essential for maintaining RBC shape, stability, and flexibility. Major components include spectrin (α, β), ankyrin (ANK1), band 3 (SLC4A1), protein 4.1, 4.2 (EPB42), and actin. Defects in these proteins cause membrane instability and premature RBC clearance. Red cell membranopathies represent a diverse group of inherited haemolytic disorders caused by defects in the erythrocyte membrane and its cytoskeletal network, affecting the structural integrity, deformability, and durability of red blood cells (RBCs). The major inherited disorders of the red cell membrane include Hereditary Spherocytosis (HS), Hereditary Elliptocytosis (HE), Hereditary Pyropoikilocytosis (HPP), and Hereditary Stomatocytosis (HSt) (Delaunay, 2007), with HS being the most common. (Perrotta et al., 2008; Narla and Mohandas, 2017).

Hereditary Spherocytosis (HS) is characterized by the presence of spherocytes—spherical RBCs lacking the central pallor—on peripheral blood smear. The pathogenesis involves defective vertical interactions between the cytoskeletal network and the lipid bilayer, primarily due to quantitative or qualitative defects in membrane proteins such as ankyrin, spectrin, band 3, and protein 4.2. These defects cause membrane loss, surface area reduction, and increased mean corpuscular haemoglobin concentration (MCHC), producing osmotically fragile, poorly deformable RBCs. These cells are sequestered and destroyed in the spleen, leading to hemolysis and shortened RBC lifespan (Barcellini et al., 2011; Da Costa et al., 2013). The clinical spectrum of HS is broad, ranging from asymptomatic carriers with compensated hemolysis to severe, transfusion-dependent anaemia. Typical features include anaemia, reticulocytosis, Jaundice, splenomegaly, indirect hyperbilirubinemia, gallstone disease, and scleral icterus. Based on clinical severity, the British Committee for Standards in Haematology (BCSH) guidelines, HS severity is categorized as trait, mild, moderate, or severe (Bolton-Maggs et al., 2012).

The five major genes implicated underlying HS are ANK1 (ankyrin), SPTA1 (α-spectrin), SPTB (β-spectrin), SLC4A1 (band 3), EPB42(protein 4.2) (Eber et al., 1996; Hassoun et al., 1996; Jarolim et al., 1996; Bouhassira et al., 1992). The inheritance is predominantly autosomal dominant (75%), although autosomal recessive (25%) forms are also observed. Additionally, *de novo* mutations have been reported in several cases. The mutation spectrum includes missense, nonsense, frameshift, splice-site variants, insertions, deletions, and duplications. These mutations are known to impair protein expression, alter binding interactions, or destabilize the cytoskeleton-membrane complex (Perrotta et al., 2008).

HS has been reported across all racial and ethnic groups, although its prevalence varies considerably among different populations. HS is particularly common in Northern Europe and North America, with an estimated prevalence of approximately 1 in 5,000 individuals in Caucasian populations (Morton et al., 1962; Gallagher, 2013; Iolascon et al., 2019). In the United States, the prevalence has been reported to be approximately 1 in 2,500 individuals (Gallagher, 2013). However, because mild forms of HS often require specialized laboratory investigations for diagnosis, the true prevalence may be four-to five-fold higher than currently reported (Eber et al., 1992; Haüser et al., 2023).

Among Asian populations, the prevalence and molecular spectrum of HS differ significantly. In China, the estimated prevalence is approximately 1 in 150 men and 1 in 800 women (Wang et al., 2015; Wang et al., 2021). Japan has reported one of the highest frequencies of HS among Asian countries, with complete protein 4.2 deficiency, inherited in an autosomal recessive manner, being relatively common compared with other populations (Yawata et al., 2000). The genetic basis of HS also exhibits marked geographic and ethnic variability. Studies from the United States have reported SPTA1 mutations as the most prevalent, followed by SPTB mutations (Agarwal et al., 2016). In contrast, studies from the Netherlands identified ANK1 mutations as the most common genetic defect, followed by SPTA1 mutations (van Wijk, 2015). Similarly, studies in Korean patients demonstrated that ANK1 mutations were the predominant cause of HS, followed by SPTB mutations, whereas SPTA1 mutations were relatively uncommon, highlighting differences from Caucasian populations (Park et al., 2016).

Globally, mutations in ANK1, SPTB, SLC4A1, SPTA1, and EPB42 are responsible for the majority of HS cases. Earlier studies from the United States and Europe estimated that these genes account for approximately 60%, 10%, 15%, 10%, and 5% of HS cases, respectively (Eber and Lux, 2004; Perrotta et al., 2008). More recent molecular studies across diverse populations, including those from the United States, Europe, Brazil, Japan, and Korea, have shown that ANK1 mutations account for nearly 50% of cases, followed by SPTB (∼20%), SLC4A1 (∼15%), EPB42 (∼10%), and SPTA1 (∼5%) mutations (Wu et al., 2021). These geographical differences suggest the existence of population-specific mutation hotspots or founder effects. Recently, a comprehensive understanding of the HS genetic spectrum for accurate diagnosis, optimized personalized management, and the eventual development of gene-based therapeutic interventions has been published by a group of researchers from Iraq population study (Abdulhassn et al., 2026).

The solute carrier family 4 member 1 (*SLC4A1*) gene (OMIM+109,270, NM_000342.4), spanning approximately 20 kb, is located on chromosome 17q21.31. *SLC4A1* encodes two protein isoforms: the Cl^−^/HCO3^−^anionicexchange transporter 1 in erythrocytes (band 3/eAE1) and a truncated form of this anionic exchange transporter 1, found in α-intercalated cells of the kidney (kAE1). Approximately 33% of HS patients exhibit a deficiency of the band-3 protein, presenting with mild to moderate clinical symptoms and a dominantly inherited pattern (Perrotta et al., 2008). In contrast, patients with compound heterozygous or homozygous band-3 defects tend to show a more severe disease phenotype (Ribeiro et al., 2000; Bracher et al., 2001; Bruce et al., 1998; Perrotta et al., 2005).

The primary mutations in the *SLC4A1* gene are missense and frameshift variants, distributed across different regions of the gene (Jarolim et al., 1996; Gallagher, 2004; Delaunay, 2007). Research linking *SLC4A1* mutations to HS indicates that most occur in exons, potentially resulting in a truncated or unstable band3protein, which impairs its function. Moreover, mutations can arise in introns, resulting in abnormal mRNA processing or a premature termination codon (Sánchez-López et al., 2010; Van Zwieten et al., 2013).

Mutations in the fifth outer loop of the band 3 protein’s transmembrane domain are associated with features of dehydrated hereditary stomatocytosis or cryohydrocytosis (Stewart et al., 1996). Due to this, a continuum is observed in the clinical presentation of patients with band-3-related hereditary spherocytosis, ranging from dehydrated hereditary stomatocytosis to cryohydrocytosis (Tanner, 2002). Mutations in the *SLC4A1* gene in humans can give rise to two distinct conditions: HS and distal renal tubular acidosis (dRTA) (More and Kedar, 2021). These disorders arise from the loss of erythrocyte membrane deformability and impaired urine acidification, respectively (Tanner, 2002). Some Band-3 mutations cause dRTA, with or without HS, and can also lead to conditions like Southeast Asian Ovalocytosis (SAO) and acanthocytosis (Bruce et al., 2000; Tanner, 2002). While mutations in *SLC4A1* are found in HS patients, they are not exclusive to this condition, and the possibility of dRTA, either alone or in combination with HS, should be considered. Furthermore, even in the presence of an *SLC4A1* mutation, thorough analysis of clinical data and other relevant laboratory results is essential (He et al., 2018).

To date, 75 distinct *SLC4A1* gene variants associated with the HS phenotype have been reported in the HGMD database (http://www.hgmd.cf.ac.uk/; accessed on 30 September 2025). These include 33 missense, seven nonsense, 21 small deletions, 1 small insertion, 1 gross insertion, and 12 splicing mutations. A total of 14 *SLC4A1* mutations associated with hereditary spherocytosis (HS) have been documented in the Indian population (Aggarwal et al., 2020).

Many studies across the globe have been performed on HS patients for their genetic testing by NGS and their phenotype and genotype correlation was investigated, showing a heterogeneous pattern (Choi et al., 2019; van Vuren et al., 2019; Tole et al., 2020). The arrival of recent advancements in next-generation sequencing (NGS) technology has proven to be highly reliable, faster, and cost-effective, allowing us to monitor multiple genes simultaneously. Numerous studies worldwide have utilized next-generation sequencing to conduct genetic testing on patients with HS. These studies have explored the correlation between genotype and phenotype, revealing a diverse and heterogeneous pattern amongst the cases (Agarwal et al., 2016; Aggarwal et al., 2020; Andolfo et al., 2021). The present study aims to report the molecular spectrum of *SLC4A1*-related HS in a case series of seven Indian patients, including a novel variant expanding the mutational landscape of *SLC4A1* defects causing HS using NGS technology.

## Materials and methods

### Study group

The seven unrelated patients were referred to our ICMR–National Institute of Immunohaematology (ICMR-NIIH) laboratory as part of routine clinical diagnostic evaluation for suspected hereditary haemolytic anaemia. All patients presented with clinical manifestations consistent with haemolytic anaemia and were investigated to establish a molecular diagnosis. Written informed consent was obtained from all participants and/or their legal guardians. The study protocol was reviewed and approved by the Institutional Ethics Committee of ICMR-NIIH (Approval No. ICMR-NIIH/IEC/16/2020, approved on 24th June 2020). Clinical features and family histories were documented whenever available. A total of 5 mL of peripheral blood was collected in EDTA vacutainers from each proband and available family members. All study procedures were conducted in accordance with the ethical standards of the institutional research committee and the principles of the 1964 Declaration of Helsinki and its subsequent amendments. They were not selected based on prior negative results for common enzymopathies but represented consecutive clinically suspected cases referred for diagnostic workup.

### Haematological, biochemical, and clinical assessment

Blood investigations included RBC count, haemoglobin (HGB) level, mean corpuscular volume (MCV), mean corpuscular haemoglobin (MCH), mean corpuscular haemoglobin concentration (MCHC), red cell distribution width (RDW), and reticulocyte (RET) count using an automated haematology analyzer (Sysmex K-1000, Japan). Peripheral smears were examined with Leishman’s stain. An eosin-5′-maleimide (EMA) binding assay on the Amnis ImageStream®X Mark II Imaging Flow Cytometer (IFC) assessed RBC morphology to aid in diagnosing Hereditary Spherocytosis (More et al., 2010). A complete laboratory work of the patient information regarding sex, age, clinical, biochemical, and haematological parameters conducted at the ICMR-National Institute of Immunohaematology (NIIH) during investigations are described in Table 1. Additional tests ruled out hemoglobinopathies and RBC enzymopathies following standard protocols. Genomic DNA was extracted from peripheral blood using the FlexiGene DNA Kit (Qiagen) for genetic analyses.

**TABLE 1:** Laboratory hematologic findings and clinical manifestations of HS patients associated with SLC4A1 variants.

Pt	Age/Sex	RBC (x 10^6^/µL)	Hb (g/dL)	MCV (fL)	MCHC (g/dL)	RDW (%)	Retic Count (%)	Tx^n^ Units	EMA (MFI)	Presenting symptoms	PBS	Indirect Bilirubin (μmol/L)	Phenotype
1	22Y/M	4.98	14.7	83.9	35.2	17.9	3	Once	56,831	Jaundice, pallor, icterus, splenomegaly	Spherocytes++	20.4	Mild
2	28Y/M	2.17	10	74.9	34.1	21	5	3 times	57,642	Jaundice since birth, Hepatosplenomegaly, gallstones, Underwent cholecystectomy	Spherocytes++	42.5	Mild
3	20Y/M	4.79	12.5	88	35	13.6	4	No	47,553	Intermittent jaundice, moderate splenomegaly, yellowish discoloration of eyes	Spherocytes+	62.5	Mild
4	3Y/M	3.64	6.7	85.7	34	23.7	14	2-3	54,721	Severe anaemia, weakness, splenomegaly	Spherocytes++, acanthocytes	68	Moderate
5	13Y/M	2.61	8.5	92.3	35.3	19.3	5	1	54,558	Anaemia, splenomegaly, mild jaundice	Spherocytes++	72	Mild
6	2Y/F	3.61	7.9	86.2	33.1	33.2	12	2	56,639	Mild anaemia, jaundice	Spherocytes++	51	Mild
7	3Y/M	3.01	11	87	34.4	20.9	3	2	58,462	Mild anaemia, jaundice	Normocytic, normochromic, spherocytes	25.5	Mild

Abbreviations: Pt.- patient, RBC- red blood cell, Hb- Haemoglobin, MCV- mean cell volume, MCHC- mean cell haemoglobin concentration, Tx^n^- Transfusions, EMA- *Eosin-5-maleimide, MFI- mean fluorescence intensity,* PBS-Peripheral blood smear.

Reference range: Erythrocyte count: 4.2-5.9 × 10^6^/µL, haemoglobin, Male: 14–17 g/Dl; Female: 12–16 g/dL, Children: 11.5–15.5 g/dL, MCH: 28–32 pg, MCHC:32–36 g/dL, MCV: 80-100 fL, RDW, Male: 11.8%–14.5%; Female:12.2%–16.1%, Reticulocyte count: 0.5%–1.5% of erythrocytes, EMA-MFI: 65,000-85,000 MFI, units, Indirect bilirubin: 3.4–12.0 μmol/L.

### Targeted next-generation sequencing (t-NGS)

A custom t-NGS panel (Illumina) targeted exonic and splice-site regions of 81 genes implicated in RBC membranopathies, enzymopathies, hemoglobinopathies, Congenital Dyserythropoietic Anaemia (CDA), Diamond–Blackfan Anemia (DBA), and Congenital Sideroblastic Anaemia (CSA), all known causes of congenital hemolytic anaemia (Supplementary Table S1). Libraries were prepared using Illumina TruSeq Custom Amplicon v1.5 and sequenced on the NextSeq 1000/2000 platform (2 × 150 bp), achieving >80–100× coverage, including exons, promoters, and UTRs. Following adapter removal, reads were aligned to GRCh37/hg19 with Sentieon, and variants were called via Sentieon haplotype caller (Freed et al., 2017) following ACMG guidelines (Richards et al., 2015). Annotation used ClinVar, OMIM, GWAS, HGMD, and SwissVar, with functional predictions from multiple *in silico* tools. All variants were confirmed by Sanger sequencing.

### Pathogenicity prediction

The pathogenicity of identified SLC4A1 missense variants was evaluated using multiple *in silico* prediction algorithms, including PolyPhen-2, SIFT, MutationTaster, Mutation Assessor, FATHMM, PANTHER, MutPred2, REVEL, VEST, and Combined Annotation Dependent Depletion (CADD). These tools assess the potential impact of amino acid substitutions on protein structure and function using evolutionary conservation, physicochemical differences, sequence homology, and machine-learning approaches. Grantham scores were used to quantify physicochemical differences between wild-type and substituted amino acids, while MuPro was used to predict the effect of variants on protein stability. Variants were interpreted collectively, with concordant predictions across multiple algorithms considered supportive evidence for pathogenicity (Niroula and Vihinen, 2019).

### Bioinformatics analysis

SLC4A1 variants were analyzed *in silico* using HOPE (Venselaar et al., 2010). Pathogenicity was predicted with SIFT, PolyPhen-2, and MutationTaster. Effects of amino acid substitutions on protein stability were assessed using DynaMut, calculating ΔΔG (ΔG_mutant–ΔG_wild-type); variants with ΔΔG < −1.0 kcal/mol were destabilising, >1.0 kcal/mol stabilising, and between −1.0 and 1.0 kcal/mol neutral. DynaMut also modeled novel variants to evaluate impacts on stability and interatomic interactions (Pires et al., 2014; Rodrigues et al., 2018). The reference SLC4A1 sequence was obtained from UniProtKB (P02370).

## Results

### Clinical and haematological findings

The study group included seven patients (six males and one female) aged between 2 and 28 years, diagnosed with hereditary spherocytosis (HS) associated with mutations in the SLC4A1 gene. The majority of patients presented in childhood with anaemia, jaundice, and splenomegaly, although the clinical severity varied considerably. Jaundice was the most common presenting feature, documented in all cases, while pallor, icterus, and splenomegaly were frequently observed. Hepatomegaly was reported in some patients, and one patient had a history of gallstone disease and cholecystectomy, highlighting the long-term complications associated with HS. Fatigue, weakness, and growth-related concerns were also observed in the younger age group.

Concerning the haematological parameters, the haemoglobin levels ranged widely from 6.7 g/dL to 14.7 g/dL, reflecting a spectrum from moderate anaemia to near-normal levels. Red blood cell counts varied between 2.17 ×10^6^/µL and 4.98 ×10^6^/µL, with the lowest values noted in patients requiring repeated transfusions. The mean corpuscular volume (MCV) was generally within the normal range (74.9–92.3 fL), while the mean corpuscular haemoglobin concentration (MCHC) was characteristically elevated in several patients, ranging from 33.1 to 35.3 g/dL, consistent with HS. Red cell distribution width (RDW) was markedly increased in all cases (13.6%–33.2%), reflecting significant red cell membrane fragmentation. Reticulocyte counts were elevated in all patients (3%–14%), indicating active bone marrow response to ongoing haemolysis. Indirect bilirubin was raised in every case, with levels between 20.4 and 68 μmol/L, corroborating increased red cell breakdown. This was clinically manifested by varying degrees of jaundice, ranging from intermittent mild scleral icterus to persistent yellowish discolouration.

All patients demonstrated the presence of spherocytes, ranging from moderate to numerous. In one case, acanthocytes were also noted, suggesting membrane instability and secondary red cell shape alterations. In milder cases, normocytic normochromic red cells were observed alongside spherocytes. All patients demonstrated reduced mean fluorescence intensity (MFI), confirming the membrane protein defect diagnostic of HS. The transfusion history was variable across the group. While some patients required no transfusions or only a single episode, others needed multiple transfusions (up to three episodes or repeated over time), highlighting heterogeneity in clinical severity. Elevated MCHC, increased RDW, reticulocytosis, and hyperbilirubinemia, together with the presence of spherocytes on smear and reduced EMA fluorescence, were consistent features across the group, supporting the diagnosis of HS.

Based on haematological indices, clinical presentation, and transfusion requirements, most patients were classified as having a mild phenotype, with one patient fitting into the moderate category. This moderate phenotype patient has HS co-inherited with dRTA, exhibiting nephrocalcinosis and failure to thrive requiring transfusions. None of the patients fell into the severe HS category.

### Molecular characterization and spectrum of the *SLC4A1* variants in HS patients

Targeted NGS identified one novel and six previously reported *SLC4A1* variants in seven unrelated Indian patients with HS (Table 2). Among the seven identified *SLC4A1* variants, three were classified as pathogenic and four as likely pathogenic according to ACMG guidelines, suggesting their potential role in disease manifestation. Six patients carried the *SLC4A1* variants in a heterozygous form, while one patient had compound heterozygous *SLC4A1* variants. The previously reported *SLC4A1* variant p.K56E, also known as Band 3 Memphis-II, was detected repeatedly in two patients.

**TABLE 2:** List of *SLC4A1* gene variants causing Hereditary Spherocytosis identified in our Indian cohort by t-NGS panel.

Pt. No.	*SLC4A1* Variants	Exon/Intron	Domain	Type	Zygosity	ACMG Guidelines	Novel/Reported	Affected parent
1	c.166A>G p. Lys56Glu	Exon 4	Cytoplasmic Domain	Missense	Hetero	Pathogenic	Reported	Father
2	c.166A>G p. Lys56Glu	Exon 4	Cytoplasmic domain	Missense	Hetero	Pathogenic	Reported	NA
3	c.2173A>C p. Ser725Arg	Exon 17	Bicarbonate transporter Domain (TM10)	Missense	Hetero	Likely pathogenic	Reported	Mother
4	c.1469G>A p. Arg490His	Exon 13	Bicarbonate transporter Domain (TM3)	Missense	Hetero	Likely pathogenic	Reported	Father
	c.2573C>A p. Ala858Asp	Exon 19	Bicarbonate transporter Domain (TM13)	Missense	Hetero	Pathogenic	Reported	Mother
5	c.2278C>T p. Arg760Trp	Exon 16	Bicarbonate transporter Domain (TM 10)	Missense	Hetero	Pathogenic	Reported	Mother
6	c.2105T>C p. Phe702Ser	Exon 17	Bicarbonate transporter Domain (TM 9)	Missense	Hetero	Likely pathogenic	Novel	Mother
7	c.173A>G p. Tyr58Cys	Exon 5	Cytoplasmic domain	Missense	Hetero	Likely pathogenic	Reported	Mother

Abbreviations: Hetero- Heterozygous; TM, Transmembrane.

**FIGURE 1:**
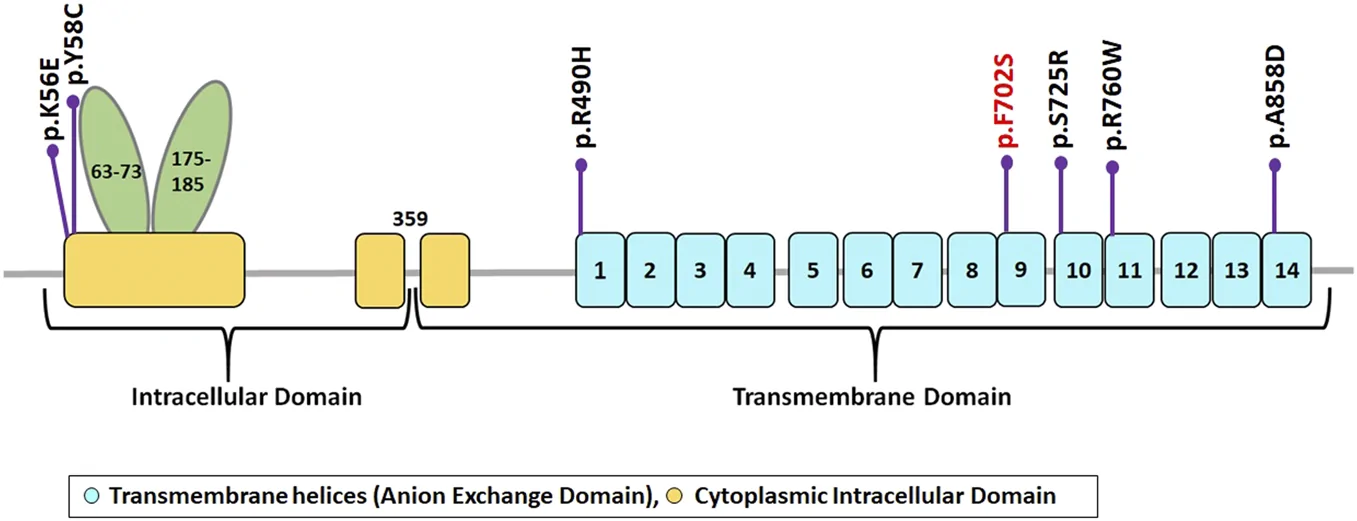
Schematic representation of *SLC4A1* variants identified in HS patients. Novel variants are depicted in red colour.

Figure 1 illustrates the structure of the *SLC4A1* gene, including the genetic map and the location of all variants concerning the domains of the SLC4A1 protein. t-NGS analysis of the *SLC4A1* gene revealed multiple missense variants distributed across the cytoplasmic intracellular domain and bicarbonate transporter domains spanning the transmembrane (TM) helices 1-14. A recurrent substitution c.166A>G (p.Lys56Glu) in exon 4 affecting the cytoplasmic domain was observed in two patients, both in a heterozygous state, classified as pathogenic and previously reported, with one case inherited from the father and the other with no parental data available. In another patient, the variant c.2173A>C (p.Ser725Arg) in exon 17 (TM10) involving the bicarbonate transporter domain was identified in a heterozygous state, reported earlier and classified as likely pathogenic, inherited maternally. Compound heterozygosity was detected in one patient with two variants: c.1469G>A (p.Arg490His) in exon 13 (bicarbonate transporter domain (TM1), likely pathogenic, paternal origin) and c.2573C>A (p.Ala858Asp) in exon 19 covering TM14 (pathogenic, maternal origin). Additionally, distinct heterozygous missense variants were found, including c.2278C>T (p.Arg760Trp) in exon 16 spanning TM11 (pathogenic, maternally inherited), and c.173A>G (p.Tyr58Cys) in exon 5 (cytoplasmic domain, likely pathogenic, maternally inherited).

A novel c.2105T>C (p.Phe702Ser) in exon 17 spans TM9 is a likely pathogenic variant inherited maternally in the proband. The novel SLC4A1 variant c.2105T>C (p.Phe702Ser) was classified according to ACMG/AMP guidelines (Richards et al., 2015). PM1 was applied as the variant is located within the functionally important transmembrane domain of Band 3 (AE1), a region enriched for pathogenic hereditary spherocytosis variants. PM2 was applied based on its absence/rarity in population databases. PP1 was assigned due to segregation of the variant with disease in the affected mother, who also demonstrated a positive EMA test and a consistent clinical phenotype. PP3 was applied as multiple *in silico* prediction tools supported a deleterious effect of the amino acid substitution. PP4 was assigned because the proband and mother exhibited a phenotype highly specific for hereditary spherocytosis, including positive EMA testing. Based on the combination of PM1+PM2+PP1+PP3+PP4**,** the variant was classified as Likely Pathogenic**.**

**FIGURE 2:**
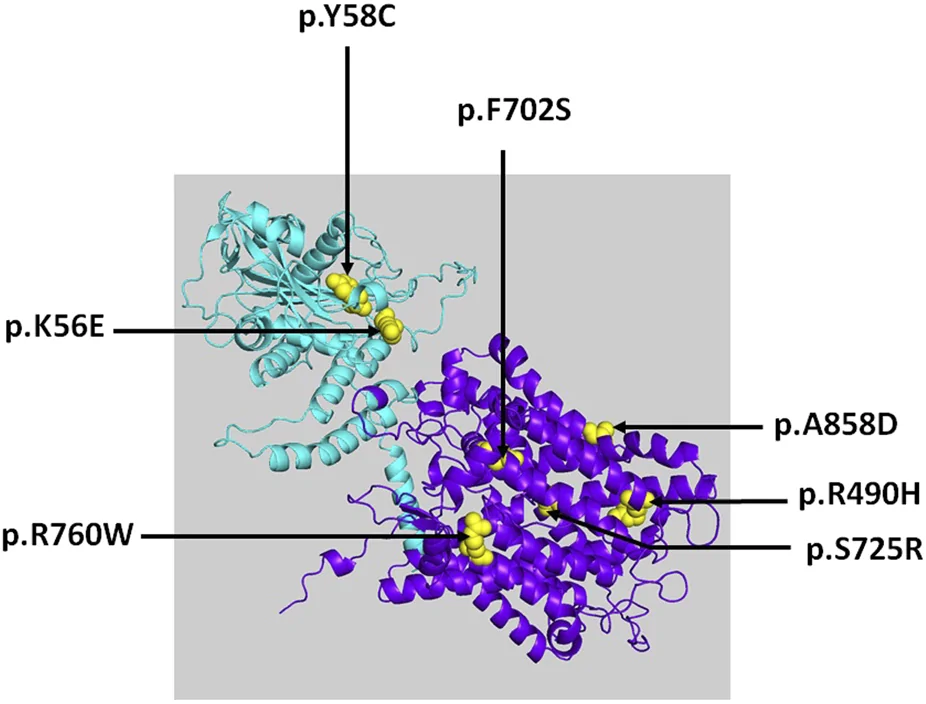
Sanger validation of the SLC4A1-HS variants identified in our cohort study.

The identified genetic variants, as determined by high-throughput analysis, were also validated by Sanger sequencing (Figure 2). Segregation analysis of all the seven patients with previously reported and novel variants has been performed and included in Supplementary Figure S1. The molecular characterization and pathogenicity of these variants were confirmed using *in silico* tools (Table 3). No additional variants were identified in other genes known to be associated with hereditary spherocytosis and hemolytic anaemia.

**TABLE 3:** Molecular characterisation of *SLC4A1*- HS patients in India and pathogenicity confirmed by various mutation prediction tools.

Pt. No.	Variants in the *SLC4A1* gene	PolyPhen 2.0	Mutation Taster	Mutation Assessor	REVEL	VEST	SIFT	FATHMM	PANTHER	MutPred2	CADD-PHRED	Grantham score	Protein stability (Mupro)
1	c.166A>G p.Lys56Glu	B (0)	B (56)	Neutral (−2.015)	0.079	0.087	T (1)	T (0.89)	B (0.02)	0.048	12.24	56	0.08 (increase stability)
2	c.166A>G p.Lys56Glu	B (0)	B (56)	Neutral (−2.015)	0.079	0.087	T (1)	T (0.89)	B (0.02)	0.048	12.24	56	0.08 (increase stability)
3	c.2173A>C p.Ser725Arg	D (0.99)	D (110)	Medium (2.975)	0.782	0.828	D (0.02)	T (−1.53)	D (0.89)	0.687	26.1	110	−0.62 (decrease stability)
4	c.1469G>A p.Arg490His	D (1.0)	D (29)	High (3.79)	0.867	0.945	D (0.0)	D (−5.72)	D (0.86)	0.9	25.9	29	−1.3 (decrease stability)
	c.2573C>A p.Ala858Asp	D (0.87)	D (126)	High (3.545)	0.699	0.961	D (0.0)	D (−3.63)	D (0.86)	0.711	25.1	126	−0.87 (decrease stability)
5	c.2278C>T p.Arg760Trp	D (0.87)	D (101)	High (4.01)	0.894	0.971	D (0.0)	D (−2.16)	D (0.89)	0.843	23.9	125	−0.43072829 (decrease stability)
6	c.2105T>C p.Phe702Ser	D (0.87)	D (155)	(High) 3.42	0.948	0.940	D (0.01)	D (−1.56)	D (0.89)	0.887	28	155	−2.2494587 (decrease stability)
7	c.173A>G p.Tyr58Cys	D (0.619)	D (194)	Medium (2.205)	0.362	0.513	D (0.02)	D (−5.11)	D (0.57)	0.539	19.27	194	−0.61 (decrease stability)

Abbreviations: B- benign, D- damaging, Tolerated.

Scores of 1 are predicted to be damaging by Polyphen2, MutPred2, PANTHER, and Mutation Taster algorithms, whereas scores of 0 are predicted to be tolerated. The SIFT, score ranges from 0.0 (deleterious) to 1.0 (tolerated). In FATHMM, a score below −1.5 indicates the variant is deleterious, while a score above 1.5 suggests it is tolerated. The distance scores published by Grantham range from 5 (tolerated) to 215 (deleterious). The CADD-PHRED, score scale ranges from 0 (potentially benign) to 48 (potentially pathogenic). The REVEL, score ranges from 0 to 1, with higher scores indicating a higher likelihood that a missense variant is pathogenic.

### Pathogenicity prediction

In silico analysis demonstrated marked differences between benign and pathogenic SLC4A1 variants (Table 3). The recurrent p.Lys56Glu variant was consistently predicted to be benign or tolerated by most algorithms, including PolyPhen-2, MutationTaster, Mutation Assessor, SIFT, FATHMM, PANTHER, MutPred2, REVEL, and VEST, and was predicted to increase protein stability by MuPro. In contrast, the variants p.Ser725Arg, p.Arg490His, p.Ala858Asp, p.Arg760Trp, p.Phe702Ser, and p.Tyr58Cys were predicted to be damaging by the majority of prediction tools. These variants showed high REVEL (0.362–0.948), VEST (0.513–0.971), and CADD-PHRED (19.27–28.0) scores, indicating a high likelihood of functional impact. Furthermore, MuPro predicted decreased protein stability for all six variants. The novel p.Phe702Ser variant demonstrated the strongest pathogenicity profile, with damaging predictions from all algorithms, a high REVEL score (0.948), an elevated CADD-PHRED score (28.0), a high Grantham distance (155), and the largest predicted decrease in protein stability (MuPro score −2.25).

### Bioinformatics analysis

**FIGURE 3:**
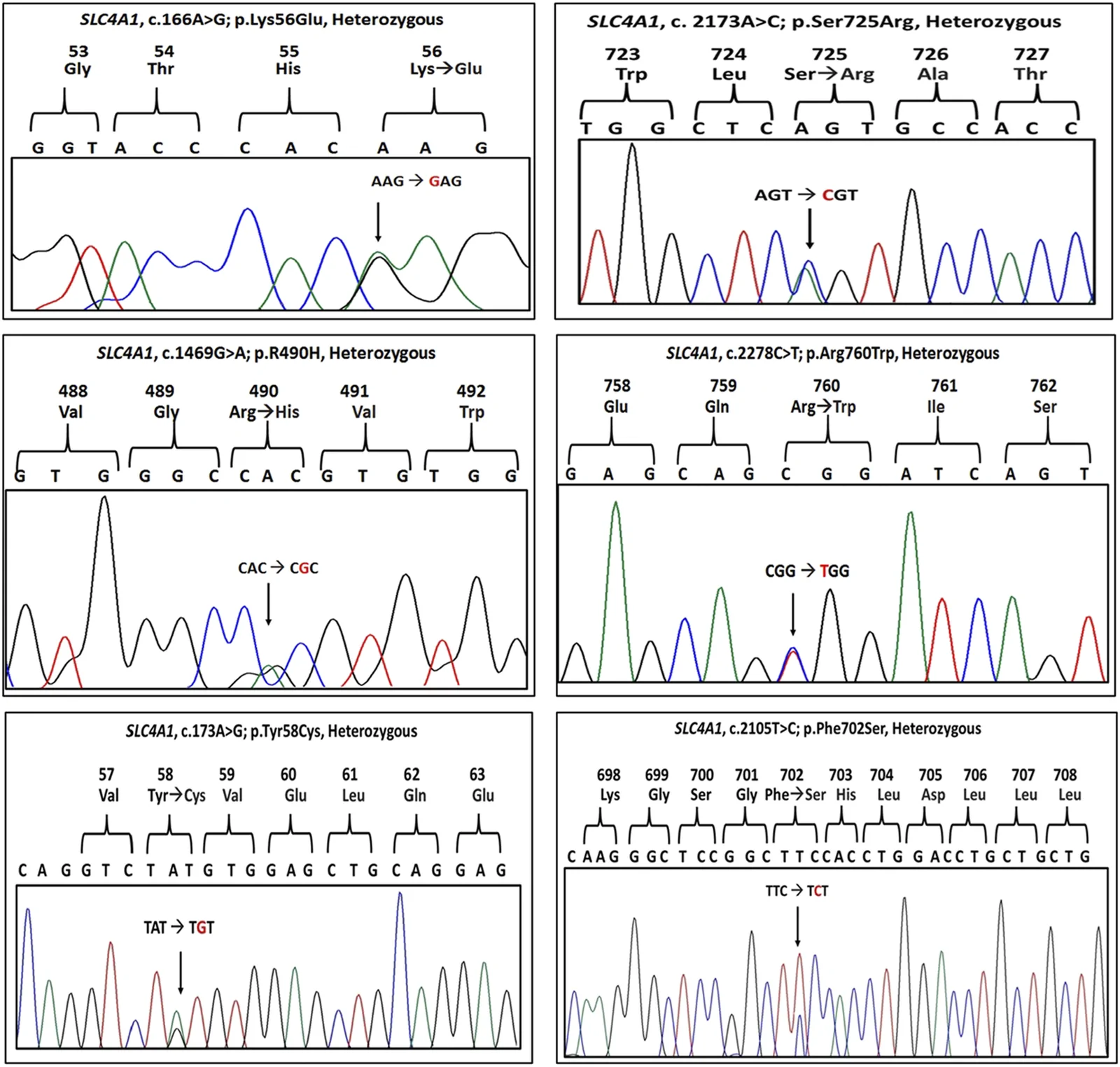
Location of SLC4A1 variants on the SLC4A1 monomer protein using PyMol.

For SLC4A1 variants, the electron cryomicroscopic structures of the human SLC4A1 protein with PDB codes 1BH7 and 4YZF were used as templates for modeling with the PyMol program. A close-up view of the identified variants on the protein structure (4YZF) was generated using PyMol, with novel variants highlighted in yellow as depicted in Figure 3.

The molecular modelling of the previously identified and reported SLC4A1 missense variants in our study cohort is shown in Supplementary Figure S2. The p.Lys56Glu (Band 3 Memphis-II) variant in the cytoplasmic domain was predicted to be benign by PolyPhen (0.0), although DynaMut suggested mild destabilization (ΔΔG = −0.209 kcal/mol) and increased flexibility (ΔΔS = +0.262 kcal·mol^-1^·K^−1^). The substitution may disrupt salt-bridge interactions with Glu22 and Asp355, potentially affecting membrane–cytoskeletal stability. The p.Ser725Arg variant in the bicarbonate transporter domain was predicted to be probably damaging (PolyPhen: 0.992). DynaMut showed a stabilizing effect (ΔΔG = +1.483 kcal/mol) but markedly reduced flexibility (ΔΔS = −1.853 kcal·mol^-1^·K^−1^). Loss of hydrogen bonding with Glu681 and altered local electrostatics may impair transporter function. Likewise, p.Arg490His was predicted to be probably damaging (PolyPhen: 1.0), with mild destabilization (ΔΔG = −0.055 kcal/mol) and increased flexibility (ΔΔS = +0.068 kcal·mol^-1^·K^−1^), likely due to loss of a salt bridge with Glu472. The p.Ala858Asp variant, located in the intramembrane region, was also predicted to be probably damaging (PolyPhen: 1.0). DynaMut indicated slight destabilization (ΔΔG = −0.020 kcal/mol) and increased flexibility (ΔΔS = +0.025 kcal·mol^-1^·K^−1^). Replacement of hydrophobic alanine with negatively charged aspartic acid may disrupt membrane interactions. The p.Arg760Trp variant was predicted to be possibly damaging (PolyPhen: 0.619), showing a stabilizing effect (ΔΔG = +0.656 kcal/mol) but reduced flexibility (ΔΔS = −0.820 kcal·mol^-1^·K^−1^), with disruption of multiple hydrogen bonds and salt bridges. Finally, p.Tyr58Cys was predicted to be possibly damaging (PolyPhen: 0.619) and showed marked destabilization (ΔΔG = −1.284 kcal/mol) with increased flexibility (ΔΔS = +0.883 kcal·mol^-1^·K^−1^), likely due to loss of hydrogen-bond interactions with Glu60 and Glu257. The pathogenicity predictions generated by multiple computational tools strongly support the deleterious nature of the identified SLC4A1 variants. Except for p.Lys56Glu, all variants were consistently predicted to adversely affect protein function and stability. The concordance among diverse prediction algorithms, including conservation-based (SIFT, PANTHER), structure/function-based (PolyPhen-2, Mutation Assessor), ensemble predictors (REVEL, VEST), and pathogenicity scoring systems (CADD, MutPred2), provides additional evidence supporting their clinical significance. Notably, the novel p.Phe702Ser variant exhibited the highest overall pathogenicity scores and the greatest predicted destabilizing effect on band 3 protein structure, consistent with its classification as a likely pathogenic variant and its association with the hereditary spherocytosis phenotype observed in the affected family.

**FIGURE 4:**
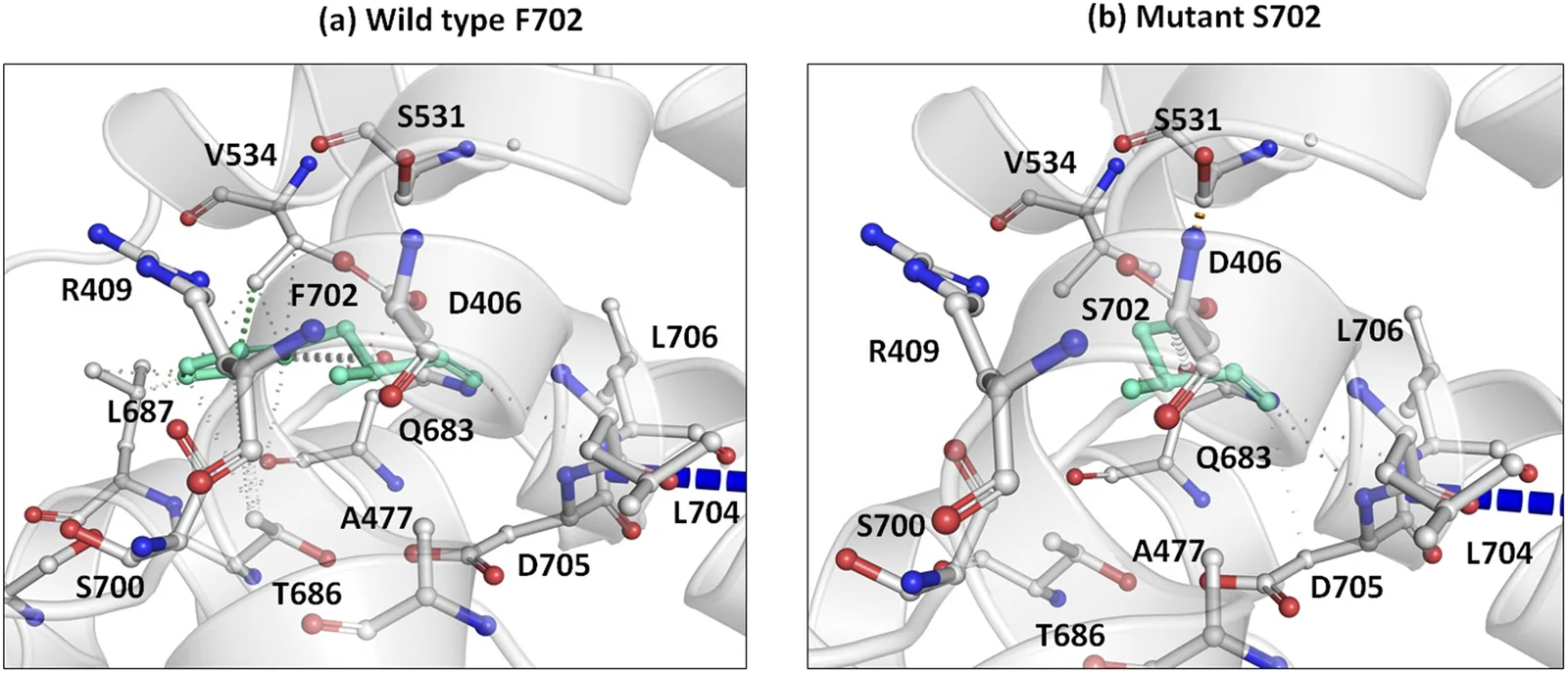
Molecular modelling of the novel missense SLC4A1 p.F702S variant using DynaMut **(a)** Wild type F701; **(b)** Mutant S702.

The p.Phe702Ser variant was predicted to be probably damaging (PolyPhen score: 0.87) and showed a strongly destabilizing effect in DynaMut (ΔΔG = −1.898 kcal/mol) with significantly increased flexibility (ΔΔS = +1.006 kcal·mol^-1^·K^−1^). The substitution of a bulky, hydrophobic phenylalanine with a polar serine disrupts key hydrophobic interactions and forms new hydrogen bonds (e.g., with Tyr393), potentially leading to improper folding and compromised the stability of the cytoskeleton in the RBC membrane (Figure 4). This variant also had a high MetaRNN score (0.961), supporting its likely pathogenic nature.

A marked variability in phenotype was observed, highlighting phenotypic heterogeneity among family members of one patient who inherited the same genotype. In Family II (Patient No.2), the father and his two children carry the same SLC4A1 variant (p.K56E); the 2 siblings showed a mild phenotype. On the other hand, the father had a transfusion history and had undergone splenectomy, while the son had cholelithiasis. Surprisingly, the daughter, however, was asymptomatic and has thus far never been transfused. Rather, the daughter was diagnosed through a family screening study.

## Discussion

Hereditary spherocytosis (HS) is the most common red cell membranopathy, caused by defects in membrane and cytoskeletal proteins such as ankyrin, spectrin, band 3, and protein 4.2 (Narla and Mohandas 2017. These defects weaken the vertical connections between the membrane skeleton and lipid bilayer, leading to membrane loss and the formation of spherocytes. Clinically, HS shows variable severity ranging from mild to severe anemia, with common features including jaundice, splenomegaly, reticulocytosis, and gallstones. Diagnosis relies on clinical and family history, physical examination, complete blood count, peripheral smear, and confirmation by the eosin-5′-maleimide (EMA) binding test (Perrotta et al., 2008; Bolton-Maggs et al., 2012).

Band 3, an anion exchange (AE1) protein in red blood cell membranes that facilitates the transport of carbon dioxide as a chloride/bicarbonate exchanger, is encoded by the *SLC4A1* gene (Jarolim et al., 1995). Studies have indicated that Band 3 deficiency occurs in about 20%–30% of HS patients in an AD manner (Perrotta et al., 2008; Bolton-Maggs et al., 2012). Approximately 15% of *SLC4A1* variants associated with HS have been reported (Wang et al., 2021). Isoforms of AE1 are expressed in both erythrocytes and the kidneys, where pathogenic variants are known to cause erythrocyte-related anemic disorders as well as distal renal tubular acidosis (Glogowska and Gallagher, 2015). We identified seven pathogenic missense variants in seven patients, distributed throughout the gene, with a Band 3 Memphis variant (p.K56E) observed repeatedly in two cases. Among the identified SLC4A1 variants, six are previously reported, and one variant is novel, causing HS. Patients with SLC4A1-HS displayed the mild to moderate phenotype.

All HS patients with *SLC4A1* variants were found to be in a heterozygous state, supporting the gene’s autosomal dominant inheritance pattern, consistent with previous findings (Perrotta et al., 2008). Reports indicate that *SLC4A1* gene defects primarily occur in exons, leading to truncated or unstable Band 3 proteins that disrupt their function. Mutations can also occur in introns, resulting in abnormal mRNA processing or truncated proteins (Jarolim et al., 1995; Van Zwieten et al., 2013). Earlier studies have demonstrated that *SLC4A1* gene defects are highly heterogeneous, located in various regions of the gene, suggesting no founder effect (Jarolim et al., 1995). In our HS cohort with *SLC4A1* defects, as in previous studies, all variants were spread throughout the gene, predominantly occurring in exons and mainly of the missense type, affecting protein function.

We identified three SLC4A1 variants in the cytoplasmic domain and four variants in the bicarbonate anion exchange domain among our HS patients. The molecular pathogenesis of Band three deficiency may vary depending on whether mutations occur in the membrane domain or the cytoplasmic domain. Mutations in the cytoplasmic domain have been shown to interfere with ankyrin binding, which is essential for transporting Band three from the endoplasmic reticulum (ER) to the Golgi apparatus for incorporation into the plasma membrane (Willardson et al., 1989; Davis et al., 1989). Thus, mutations in the cytoplasmic domain could weaken the Band 3-ankyrin interaction in the ER, leading to reduced integration of mutant Band three into the membrane (Gomez and Morgans, 1993), while mutations in the membrane binding domains result in the loss of anion exchange activity (Iolascon et al., 1992).

The previously reported SLC4A1 p.K56E variant, also known as Band 3 Memphis, was identified in two unrelated families in our cohort, is consistently classified as benign by most *in silico* prediction tools. Nevertheless, our DynaMut analysis indicates that this substitution may exert a destabilizing effect on protein conformation, suggesting a potential functional impact not captured by standard prediction algorithms. Notably, a recent case report by Shih et al., 2023 described a family from Taiwan carrying this variant who presented with clinically significant hereditary spherocytosis requiring splenectomy. Taken together, these findings highlight that variants labelled as benign should not be dismissed outright, as they may still contribute to disease in specific genetic or environmental contexts and highlight the need for periodic re-evaluation of variants in light of emerging clinical and structural data.

The novel variant p.F702S is positioned in the transmembrane helix 9 (TM9) of AE1, lies within the core subdomain of the protein and is oriented almost perpendicularly to the lipid bilayer. It faces away from the gate domain and is positioned along the outer surface of the core. TM9 contributes significantly to the structural stability of the core domain and plays an essential role in maintaining the correct conformation of the substrate-binding pocket. By stabilizing adjacent helices (TM8–11), it may indirectly influence anion binding and translocation across the membrane. Mutations affecting TM9 can disrupt the overall folding of AE1, resulting in defective anion transport or intracellular retention of the misfolded protein within the endoplasmic reticulum (Bogusławska et al., 2023). This alteration destabilized the erythrocyte membrane cytoskeleton, resulting in the manifestation of HS in the patient.

One patient had a heterozygous c.1469G>A; p. Arg490His mutation in exon 13 and a heterozygous c.2573C>A; p. Ala858Asp mutation in exon 19 of *SLC4A1*, associated with HS and dRTA. The mutation in exon 13 was inherited from his father, while the exon 19 mutation came from his mother. These findings suggest that these mutations in *SLC4A1* contribute to the combination of HS and dRTA. A similar case was reported by Chu et al. (2010), involving a TGC>TGG mutation in exon 13 and a GGC>GAC mutation in exon 17 of *SLC4A1* in a patient with HS and dRTA. Various mutations in the human *SLC4A1* gene can lead to both HS and dRTA. Since *SLC4A1* mutations are not exclusive to HS, the presence of dRTA or co-inheritance of HS and dRTA should not be neglected (Tanner, 2002). In our study, we identified one HS patient with co-inherited AR dRTA due to the *SLC4A1* p.A858D mutation. Our findings clearly indicate that, despite the presence of mutations in *SLC4A1*, a thorough analysis of clinical data and relevant laboratory results, alongside genetic testing, is essential for accurate diagnosis, family counselling, and management of these disorders.

Phenotypic variability, or genotype-phenotype discordance, which could be attributed to other modifier genes, epigenetics, incomplete penetrance, gene-environment interactions, or mosaicism has been observed in HS cases (Wang et al., 2018; Bogdanova et al., 2020). Although the underlying genetic defects in red cell membrane proteins may explain many phenotypic variations, a proportion of variability may be due to other co-inherited factors like enzymopathies, thalassemias and Gilbert syndrome. Associations of HS with HBB mutations (More et al., 2020), glucose-6-phosphate dehydrogenase (G6PD) deficiency and Gilbert syndrome (*UGTIA1* gene) in isolation have been reported previously (Jamwal et al., 2016). Nevertheless, some unknown genetic factors might also play a role in the observed phenotypic variability. Besides, non-genetic factors might influence the RBC damage and their premature removal from the circulation and increase in their heterogeneity (Bogdanova et al., 2020). Further investigation is needed to elucidate the underlying causes of phenotypic heterogeneity in HS patients, which may involve both genetic and non-genetic factors.

In our cohort, despite sharing an identical pathogenic variant, members of one family demonstrated marked genotype–phenotype discordance, reflecting underlying phenotypic heterogeneity. The observed phenotypic variability among one SLC4A1-HS patient suggests that additional genetic or environmental modifiers influence disease expression beyond the primary genotype. This variable phenotypic expression can be attributed to the alcoholism history noted in the father and the son, highlighting the modifying role of lifestyle factors on the genetic defect in disease expression.

## Conclusion

In summary, we identified seven patients with HS carrying variants in the *SLC4A1* gene using next-generation sequencing (NGS), including six previously reported and one novel pathogenic variant. The novel variant (p.F702S) within TM9 can impair the proper folding of AE1, leading to defective anion transport or accumulation of the misfolded protein within the endoplasmic reticulum precipitated the clinical onset of HS. Our findings highlight the importance of genetic testing in delineating the molecular spectrum of HS and contribute valuable insights into the genotype–phenotype correlations underlying this heterogeneous disorder. A careful evaluation of phenotypic features is essential to guide genetic diagnosis, enabling accurate identification, informed counselling, and optimal clinical management in the rare co-existence of HS and dRTA. Comprehensive molecular characterization, as demonstrated in this study, is therefore crucial for improving diagnostic accuracy and enhancing our understanding of the molecular mechanisms responsible for HS. The discovery expands the known mutational spectrum of *SLC4A1* and underscores the genetic heterogeneity underlying HS.

## Data Availability

The original contributions presented in the study are publicly available. This data can be found in the ClinVar repository with the accession number SCV007653975.

## Supplementary material

SUPPLEMENTARY FIGURE S1 Pedigree structure and segregation analysis of all the seven patients with identified SLC4A1 variants.

SUPPLEMENTARY FIGURE S2 Molecular modelling of the reported missense SLC4A1 variants using DynaMut.
