# Supplementary material for: Expanding the genetic landscape of SLC4A1-linked hereditary spherocytosis: discovery of a novel TM9 variant using high-resolution genomic profiling analysis
Source: Front Genet. 2026 Aug 27;17:1831354. doi: 10.3389/fgene.2026.1831354 (PMC13557700; doi:10.3389/fgene.2026.1831354)
Supplement: Supplementary file 1 [file Table1.docx]

Table S1: List of 81 Genes included in Targeted Next Generation Sequencing (t-NGS) Panel

| *ABCB7* | *EPB42* | *HSPA9* | *RhAG* | *SBDS* |
| --- | --- | --- | --- | --- |
| *ABCG5* | *EPB72* | *KCNN4* | *RPL11* | *SEC23B* |
| *ABCG8* | *FECH* | *KIF23* | *RPL26* | *SLC11A2* |
| *ADA* | *G6PD* | *KLF1* | *RPL27* | *SLC19A2* |
| *ADD1* | *GATA1* | *LPIN2* | *RPL35A* | *SLC25A38* |
| *AK-1* | *GCLC* | *NADH-MR* | *RPL5* | *SLC2A1* |
| *ALAS2* | *GPI* | *NT5C3A* | *RPL9* | *SLC4A1* |
| *ALDOA* | *GPX1* | *PFK-M* | *RPS10* | *SPTA1* |
| *ANK1* | *GSR* | *PGD* | *RPS17* | *SPTB* |
| *BPGM* | *GSS* | *PGK1* | *RPS19* | *STEAP3* |
| *C15orf41* | *GYPA* | *PIEZO1* | *RPS24* | *TERC* |
| *CDAN1* | *GYPC* | *PIEZO2* | *RPS24* | *TINF2* |
| *CECR1* | *HBA1* | *PIGA* | *RPS26* | *TMPRSS6* |
| *DKC1* | *HBB* | *PIGT* | *RPS27* | *TPI1* |
| *ENO1* | *HK1* | *PKLR* | *RPS7* | *TRNT1* |
| *EPB41* | *HMOX1D* | *PUS1* | *RTEL1* | *UGT1A1* |
| *YARS2* |  |  |  |  |
